# Recognition of protein/gene names from text using an ensemble of classifiers

**DOI:** 10.1186/1471-2105-6-S1-S7

**Published:** 2005-05-24

**Authors:** GuoDong Zhou, Dan Shen, Jie Zhang, Jian Su, SoonHeng Tan

**Affiliations:** 1 Institute for Infocomm Research, 21 Heng Mui Keng Terrace, 119613, Singapore; 2 School of Computing, the National Univ. of Singapore, 119610, Singapore

## Abstract

This paper proposes an ensemble of classifiers for biomedical name recognition in which three classifiers, one Support Vector Machine and two discriminative Hidden Markov Models, are combined effectively using a simple majority voting strategy. In addition, we incorporate three post-processing modules, including an abbreviation resolution module, a protein/gene name refinement module and a simple dictionary matching module, into the system to further improve the performance. Evaluation shows that our system achieves the best performance from among 10 systems with a balanced F-measure of 82.58 on the closed evaluation of the BioCreative protein/gene name recognitiontask (Task 1A).

## Background

With an overwhelming amount of textual information in biomedicine, there is a need for effective and efficient literature mining and knowledge discovery that can help biologists to gather and make use of the knowledge encoded in text documents. For example, MEDLINE [1], the primary research database serving the biomedical community, is an online bibliographic source of citations and abstracts dating from 1966 till present and currently contains over 12 million abstracts with 60,000 new abstracts each month. There are also a few molecular biological databases covering various information on genes, proteins, nucleotide and amino acid sequences, both generally (e.g. the protein sequence database SwissProt [2] and the genetic sequence database GenBank [3]) and for particular species (e.g. FlyBase [4] on the genetics and molecular biology of Drosophila). Each of them contains entries ranging from thousands to millions and multiplies rapidly. Normally, all of these resources are annotated manually by human experts. However, such manual handling is much throughput-limited, extremely time-consuming and enormously expensive. In order to make organized and structured information available, automatically recognizing biomedical names becomes critical and is important for protein-protein interaction extraction, pathway construction, automatic database curation, etc.

Such a technique, called named entity recognition, has been well developed in the Information Extraction literature [5,6]. In MUC, the task of named entity recognition is to recognize the names of persons, locations, organizations, etc. in the newswire domain. In the biomedical domain, we care about entities like genes, proteins, viruses, etc. In recent years, many explorations have been done to port existing named entity recognition systems into the biomedical domain [7-10]. However, few of them have achieved satisfactory performance due to the special characteristics of the biomedical names, such as long and descriptive naming convention, conjunctive and disjunctive structure, causal naming convention and rapidly emerging new biomedical names, abbreviation, and cascaded construction [9,10]. On all accounts, we can say that the entity names in the biomedical domain are much more complex than those in the newswire domain.

## Methods

In the competition, an ensemble of classifiers is proposed to recognize the protein/gene names, in which three classifiers, one Support Vector Machine (SVM) and two discriminative Hidden Markov Models (DHMMs), are effectively combined. In literature, various strategies have been used to integrate multiple classifiers into an ensemble, e.g. bootstrapping [11] and boosting [12]. Here, the ensemble is constructed using a simple majority voting strategy. Among the three classifiers, the only difference between the two DHMMs comes from the part-of-speech (POS) features, which are trained on different corpora. The main reason for integrating these three classifiers as an ensemble is the finding during our investigation that they have quite different characteristics with regard to the precision and recall. Our evaluation on the dry-run data shows that the SVM using the POS feature trained on the refined BioCreative-POS corpus (Please see below for details) has high precision and low recall, the DHMM1 using the POS feature trained on the refined BioCreative-POS corpus has balanced precision and recall, and the DHMM2 using the POS feature trained on the unrefined BioCreative-POS corpus has low precision and high recall. The reason that DHMM1 performs differently to DHMM2 may be due to that the refined BioCreative-POS corpus restricts much more on the NNP POS and has much less NNP POS-tagged words than the unrefined BioCreative-POS corpus. Therefore the refined BioCreative-POS tagger has much better precision and worse recall on the NNP POS, which are critical in recognizing protein/gene names, than the unrefined BioCreative-POS tagger. Such differences among SVM, DHMM1 and DHMM2 mean that they complement each other, and show the potential for significant performance improvement via an ensemble.

In addition, we also incorporate three post-processing modules: an abbreviation resolution module, a protein/gene name refinement module and a simple dictionary matching module, into the system to further improve the performance.

In this section, we will first introduce the various features used in the competition, then the two machine learning approaches and finally the three post-processing modules.

### Feature representation

The features described here are used in all the evaluations. In the competition, various features, including the surface word itself, are applied to capture the special characteristics of protein/gene names:

• **Orthographic feature**: The purpose of this feature is to capture capitalization, digitalization and other word formation information. This feature has been widely used in the biomedical domain [7,9,10]. Table 1 shows a complete list for this feature in the descending order of priority.

• **Part-of-speech (POS)**: Since many of the words in biomedical names are in lowercase, capitalization information in the biomedical domain is not as evidential as that in the newswire domain. Moreover, many biomedical names are descriptive and very long. Therefore, POS may provide useful evidence about the boundaries of biomedical names. In this competition, a DHMM [13]-based POS tagger is trained to assign the POS feature. Throughout the competition, three different POS taggers are trained on different corpora:

• GENIA-POS tagger, which is trained on the GENIA corpus V3.02p.

• Unrefined BioCreative-POS tagger, which is trained on the original BioCreative-POS corpus with the NEWGENE (indicating a protein/gene name) tag replaced by the NNP tag (indicating a proper noun).

• Refined-BioCreative-POS tagger, which is trained on a refined version of the BioCreative-POS corpus. In the competition, the refined BioCreative-POS corpus is created as follows: First, the unrefined BioCreative-POS tagger is trained as above; Second, the words inside the protein/gene names of the BioCreative-POS corpus are retagged using the BioCreative-POS tagger while the POS tags of the words outside the protein/gene names are kept unchanged; Third, the words in the protein/gene names are fine-tuned to have the NNP tag when they have a high probability of being a proper noun, e.g. when they are the head nouns of the protein/gene names or include both alphas and digits.

• **Morphological pattern**: Suffixes, such as ~ase, ~zyme, ~ome and ~gen, occur frequently in protein/gene names and are considered as an important cue for terminology identification and have been widely applied in the biomedical domain [7-10]. To reduce possible noise, some common words (58 in the competition) of these suffixes, such as *disease*, *base*, case and *come*, are filtered out.

• **Trigger word**: The head noun of a noun phrase often describes the function or the property of the noun phrase. In this paper, we automatically extract unigram and bigram head nouns from the context of protein/gene names in the training data as trigger words. In the competition, two kinds of trigger words are used: *TW1*, which often occurs inside protein/gene names, and *TW2*, which often occurs in the local context of protein/gene names. *TW1*, such as *receptor*, *enhancer and mutant*, is collected based on the *Task 1A Guideline*. *TW2*, such as *activation*, *transcription and stimulation*, is extracted automatically from the training data using the tf-idf weighing scheme [14] to measure how specific a given trigger word is to protein/gene names. Here, the tf-idf value is used in the feature vector of the SVM. In the competition, 53 *TW1 *and 51 *TW2 *trigger words are used.

### Support Vector Machine

Support Vector Machine (SVM) is a powerful machine learning method, which has been applied successfully in biomedical name recognition [7,8]. SVM is a binary classifier and training a SVM classifier is to find the optimal hyper-plane that separates positive and negative data with the maximum margin. Normally, a window of a target word *w *represents the local context of *w *and is used to make a decision on *w*. In this competition, we set the window size to 7, which includes the previous 3 words and the next 3 words of the target word *w *including the target word *w *itself. Here, each instance in the training and test data is represented using a high-dimensional feature vector. For example, if a word occurs in the vocabulary (collected from the training data), one dimension in the feature vector of the SVM (corresponding to the position of the word in the vocabulary) is set to 1. The vocabulary is constructed by taking all the words in the training data (filtered with threshold 3). In our system, all the five features as described above are applied for each of the 7 words in the window. When a word contains dash(es), one additional overlapping orthographic feature is generated for each segment separated by dashes. For example, the word "TCF-Beta" has not only an orthographic feature of "CapMixAlpha" as a whole but also two additional overlapping orthographic features "AllCaps" and "GreekLetter" for the two segments separated by the dash while the word "TCF" only has an orthographic feature of "AllCaps". Please see Table 1 for details about the orthographic feature. In the competition, we adopt the SVMLight toolkit [15] using a polynomial kernel with degree = 2.

Since there is only one name class (NEWGENE) in the BioCreative protein/gene name recognition task, we simplify the traditional *BIO *representation and employ *IO *tags to represent the regional information of protein/gene names. In this *IO *representation, *I *means that current word is a part of a protein/gene name, which corresponds to the SVM output 1; *O *means that current word is not a part of a protein/gene name, which corresponds to the SVM output -1. After the simplification, the protein/gene recognition task becomes a binary classification task. Although the *IO *representation cannot differentiate consecutive names, it simplifies the problem a lot since we can avoid the SVM multi-class problem. We find it is a worth trade-off since very few (<0.5%) protein/gene names are consecutive.

### Discriminative Hidden Markov Model

Here, we use the discriminative Hidden Markov Model (DHMM) which was first proposed in Zhou et al [13] and then applied in Shen et al [9] and Zhou et al [10]. Given an observation sequence  = *o*_1_*o*_2 _*o*_*n*_, the DHMM finds a stochastic optimal tag (state) sequence  = *t*_1_*t*_2 _*t*_*n *_that maximizes  as follows:



The above model consists of two models: the state transition model  and the output model . Here, *PMI*(*s*_*i*_, ) measures the state dependence of a state given the previous states in a generative way using pairwise mutual information [16], which reflects the change of the information content when *s*_*i *_and  co-occur, while log*P*(*s*_*i *_| ) measures the observation dependence of a state given the observation sequence in a discriminative way. Therefore, computation of the above model consists of two parts. The first is to compute the state transition model: . Here, the traditional ngram modeling (e.g. trigram) is used. The second is to estimate the output model: . Here, a dynamic back-off modelling [10] is applied.

In the competition, only three features are used in the DHMM: the orthographic feature, the POS feature and the surface word as described above. All the three features are combined and become an observation of DHMM while each tag is structural and consists of three parts [13]:

• **Boundary category **{B, I, E, O}, which indicates the position of the word. Here O means that current word is a whole name and B/I/E means that current word is at the Beginning/in the Intermediate/at the End of a name.

• **Entity category, **which indicates whether the word locates inside or outside a protein/gene name.

• **Word feature**, which is added to represent the state transition model more accurately.

The idea behind the model is that we try to assign each word an appropriate tag, which contains boundary and class information. For example, "TCF 1 binds stronger than NF kB to TCEd DNA". The tag assigned to the word "TCF" should indicate that it is at the beginning of an entity name and it belongs to the "Protein" class; and the tag assigned to the word "binds" should indicate that it does not belong to an entity name. Here, the Viterbi algorithm [17] is implemented to find the most likely tag sequence.

### Abbreviation resolution

In the competition, we present an effective and efficient algorithm to resolve abbreviations accurately by mapping them to their full forms. It is observed [9,18] that the full form and its abbreviation often occur together via parentheses. Generally, there are two patterns: "full form (abbreviation)" and "abbreviation (full form)".

Our algorithm is based on the fact that it is much harder to classify an abbreviation than its full form. Generally, the full form is more evidential than its abbreviation to determine its class. The algorithm works as follows: Given a sentence with parentheses, we use a similar algorithm as in Schwartz et al [18] to determine whether it is an abbreviation with parentheses. This is done by starting from the end of both the abbreviation and the expanded form, moving from right to left and trying to find the shortest expanded form that matches the abbreviation. Any character in the expanded form can match a character in the abbreviation with one exception: the match of the character at the beginning of the abbreviation must match the first alphabetic character of the first word in the expanded form. If yes, we remove the abbreviation and the parentheses from the sentence. After the sentence is processed, we restore the abbreviation with parentheses to its original position in the sentence. Then, the abbreviation is classified as the same class of the full form, if the full form is recognized as an entity name. In the meanwhile, we also adjust the boundaries of the full form according to the abbreviation, if necessary. In this way, we can correct the boundary error of the full form which defines an abbreviation. In addition, we can classify the abbreviation according to the prediction of its full form, since we assume it is more accurate to classify the full form than the abbreviation.

### Name refinement

In order to further improve the performance, we also develop a name refinement module. This module applies some heuristic rules to refine the recognized protein/gene names according to the Task1A guidelines and recover the errors caused by the inconsistency and the improper tokenization in the training data, e.g.

• Extending recognized names by adding positive trailer words. For example, If only "p53" in "p53 mutant" is recognized as a protein/gene name, we will add "mutant" into the name since "mutant" is a positive trailer word. Similarly, we also shorten the recognized names by removing negative trailer words. This rule is very general. It suggests that we need to model the ends of biomedical names.

• Removing generic adjective words, e.g. "new" and "novel" in the beginning of recognized protein/gene names. This rule can compensate the failure of IO representation in the SVM to model the beginnings of biomedical names, compared with the BIO representation, even though the IO representation simplifies the problems and makes the data denser.

• Recovering errors caused by the wrong tokenization of "." in the recognized protein/gene names, e.g. "UL3 **. **5" and "E **. **coli RNase H". This rule is quite corpus specific. It deals with the special tokenization scheme used in the BioCreative annotation.

• Removing stop words, e.g. "by" and "or", from the recognized names, which have been wrongly recognized as a part of the names.

• Formalizing the recognition of slash and parentheses

• Removing generic terms (all the words used in the name are too common), e.g. "protein kinase".

• Removing odd names, such as individual digits and Greek letters.

### Dictionary matching

Finally, we also evaluate the effectiveness of a protein/gene name dictionary using a simple dictionary matching algorithm. The basic assumption behind and motivation for using public resources is that there are instances where the contexts do not provide sufficient evidence. In such cases, public resources, e.g. a protein/gene name dictionary, may bridge the gap. In our closed evaluations, the dictionary is constructed by extracting all protein/gene names from the training data. In our open evaluation, the dictionary adds more protein/gene names (~700,000 entries) from public resources (e.g. the protein sequence database [2] and the alias list [19]). Then, the dictionary is filtered out using some criteria to reduce possible side-effects since all the public resources are vulnerable to noise and ambiguity. For example, if a name consists of only one word, the length of the word must be greater than 3. Finally, we use the dictionary to match the test data and correct the output of the ensemble.

## Results and discussion

In the BioCreative competition, we only participated in the protein/gene name recognition task (Task 1A), focusing on the closed evaluation. In total, we submitted three closed evaluations and one open evaluation. The final system is trained on the combined official training and dry-run data (10000 sentences). All the open and closed evaluations are done on the official test data (5000 sentences) using the precision/recall/F-measure. Here, precision (P) measures the number of correct protein/gene names in the answer file over the total number of protein/gene names in the answer file, recall (R) measures the number of correct protein/gene names in the answer file over the total number of protein/gene names in the key file and F-measure is the weighted harmonic mean of precision and recall:  with β^2 ^= 1 [20]. The evaluation shows that our system on the closed evaluation performs the best out of all the closed systems with an F-measure of 82.58, which is 0.4 and 2.2 higher than the second and third best systems. It also shows that our closed system performs only slightly worse (0.6) than the best open system and better than other open systems. It is surprising because we had expected that the best open system should outperform the best closed system by at least 2–4 in the F-measure. One major reason is due to our use of the classifier ensemble and the effective post-processing modules, abbreviation resolution and name refinement (as shown in Table 3). Another reason may be somewhat corpus specific. For example, one system-component that seems to help our score quite a bit (as shown in Table 3) is the name refinement module, which is built in part to adjust decisions to conform more to what is marked in this corpus. Finally, it may be due to the difficulty in exploring public resources and the subtlety about what does and what does not count as a protein/gene name in biomedical name recognition. This suggests that exploring public resources still remains a big problem and much more research should be made in this direction in the near future.

Table 2 indicates the performances and configurations of all the closed and open evaluations. For clarity, the differences and their contributions among different evaluations are emphasized (in Bold, compared with the left configuration). It shows that the POS tagger trained on the refined version of the BioCreative-POS corpus works better (+0.40) than the POS tagger trained on the GENIA corpus V3.02p (closed-2 vs. closed-1). This may be because the refined BioCreative-POS corpus is more task-oriented than the GENIA corpus V3.02p, although the GENIA corpus V3.02p comes from the biomedical domain and is larger than the BioCreative-POS corpus (360 k words vs. 260 k words). It also shows that the protein/gene name refinement module increases the F-measure by 2.35 (closed-3 vs. closed-2). However, the open evaluation shows that extra protein/gene names from public resources decrease the performance by 2.57 in F-measure from 80.63 to 78.06 (closed-2 vs. open-1). This is largely due to the short time spent on the open evaluation (half day). This may be also due to the high ambiguity in the open dictionary of protein/gene names. This suggests that proper handling of public knowledge resources is important for the performance improvement in biomedical name recognition.

Table 3 shows the detailed performance of various components in our best closed evaluation (closed-3). It shows that individual SVM, DHMM1 and DHMM2 achieve the precision/recall/F-measure of 75.1%/70.2%/72.7, 71.6%/71.9%/71.8 and 70.1%/74.3%s/72.1 respectively on the official test data. This means that these three classifiers are quite complementary (some have better recall, others have better precision) and this provides a potential for further performance improvement via an ensemble. This is proven by the ensemble of these three classifiers via a simple majority voting strategy, which improved the F-measure by about 3.7 (over SVM). It also shows that the abbreviation resolution and name refinement modules further improve the F-measure by 3.7 and 2.4 respectively. Finally, the dictionary matching module using the closed dictionary only slightly improves the F-measure by 0.5. It increases the recall by 1.5% but decreases the precision by 0.4%. While the abbreviation resolution is very general, the name refinement and the dictionary matching are fine-tuned based on the error analysis of the development set and may be very much dependent on the development set.

Table 4 shows the contributions of various features in our best closed evaluation (closed-3). It measures the decrease in precision/recall/F-measure by leaving one feature out at a time. It shows that the orthographic feature, POS and surface word are critical and contributes about 96% of all the features while the remaining morphological feature and trigger word only contribute about 4%.

Finally, in order to further evaluate our system, we have implemented an error analysis. This is done by randomly choosing 100 errors from our recognition results, which can be classified as follows:

• **Left boundary errors **(37): It includes the errors with correct right boundary detection and only wrong left boundary detection. We find that most of such errors come from the long and descriptive naming convention in the biomedical names. In fact, it is even hard for biologists to decide whether the descriptive words should be a part of protein/gene names, such as "*normal", "activated"*, etc.

• **Right boundary errors **(9): It includes the errors with correct left boundary detection and only wrong right boundary detection. It usually occurs when the head words of the protein/gene names seldom occur in the training data and the system fails to model these less-frequently occurring head words.

• **True negative **(23): It includes the errors by missing the identification of protein/gene names. It often occurs when the system has little information about them and context clues are insufficient.

• **False positive **(22): It includes the errors by wrongly identifying protein/gene names. It usually occurs when a noun phrase is a description of a protein/gene name or includes symbols or is mixed with digits and capitalized letters.

• **Miscellaneous **(9): It includes all the other errors, mostly caused by parentheses and the tokenization scheme in the BioCreative annotation (e.g. the dot "."). Although we have applied a name refinement module to filter such errors, it is always difficult to cover special cases.

## Conclusion

In this paper, we first propose an ensemble of classifiers for biomedical name recognition via a simple majority voting strategy to effectively integrate various domain-specific features and then present several ad-hoc post-processing modules to further improve the performance. It is found that the integrated three classifiers are quite complementary and the ensemble of them via the simple majority voting strategy can greatly improve the performance. Moreover, it shows that our system benefits very much from the post-processing modules, such as abbreviation resolution and name refinement. Finally, it shows that the dictionary matching module using a very big open dictionary decreases the performance. This means that the improper use of knowledge resources is harmful to biomedical name recognition.

In future work, we will explore more classifiers and more effective approaches to integrate them in an ensemble. Moreover, we will explore proper approaches for handling the large open dictionary and even more knowledge resources. Finally, we will further improve the performance by investigating more on conjunctive and disjunctive construction, the synonym phenomenon and the name alias phenomenon.

## Availability

Technology license is available with a bilateral agreement. Please contact Dr. Su Jian (the 4^th ^author SJ: sujian@i2r.a-star.edu.sg) for details.

## Authors' contributions

ZGD carried out the studies in DHMMs and the majority voting strategy, took in-charge of the whole system and drafted the manuscript. SD implemented the SVM, the abbreviation resolution and dictionary matching module. ZJ implemented the SVM, the abbreviation resolution and name refinement modules. SJ initiated the multi-classifier approach, organizedand coordinated the above efforts. TSH gave feed-back on biomedical name boundaries and tokenization. All authors have read and approved the final manuscript.

## Figures and Tables

**Table 1:** Orthographic Feature

Features 1–11	*e.g.*	Features 12–21	*e.g.*
Comma	*,*	OneCap	*T*
Dot	*.*	AllCaps	*CSF*
Parenthesis	*() []*	CapLowAlpha	*All*
RomanDigit	*II*	CapMixAlpha	*IgM*
GreekLetter	*Beta*	LowMixAlpha	*kDa*
StopWord	*in, at*	AlphaDigitAlpha	*H2A*
ATCGsequence	*ACAG*	AlphaDigit	*T4*
OneDigit	*5*	DigitAlphaDigit	*6C2*
AllDigits	*60*	DigitAlpha	*19D*
DigitCommaDigit	*1,25*	Others	*Other*
DigitDotDigit	*0.5*		

**Table 2:** Performance and configurations of all the evaluations in the protein/gene name recognition task

Modules	Closed-1	Closed-2	Closed-3	Open-1
SVM	Surface word, orthographic feature, morphological pattern, trigger word			
	GENIA-POS	**Refined-BioCreative-POS**	Refined-BioCreative-POS	Refined-BioCreative-POS

DHMM1	Surface word, orthographic feature			
	GENIA-POS	**Refined-BioCreative-POS**	Refined- BioCreative-POS	Refined-BioCreative-POS

DHMM2	Surface word, orthographic feature, BioCreative-POS			

Ensemble	Majority Voting			

Abbreviation Resolution	Abbreviation Resolution based on the parentheses structure			

Name Refinement	N/A	N/A	**YES**	N/A

Dictionary Matching	Closed Dictionary	Closed Dictionary	Closed Dictionary	**Open Dictionary**

Overall Performance	P79.97	P80.46	P82.00	P75.10
	R80.50	R80.80	R83.17	R81.26
	F80.23	F80.63(**+0.40)**	F82.58(**+2.35)**	F78.06(**-4.52)**

**Table 3:** Detailed performance of various components in our best closed system (closed-3)

Configuration	P	R	F
SVM (individual)	75.1	70.2	72.7
DHMM1 (individual)	71.6	71.9	71.8
DHMM2 (individual)	70.1	74.3	72.1
Ensemble (majority voting)	75.9	77.0	76.4
Ensemble + Abbreviation Resolution	79.8	80.4	80.1
Ensemble + Name Refinement	78.6	79.1	78.8
Ensemble + Dictionary Matching	75.5	78.5	76.9
All (overall performance)	82.0	83.2	82.6

**Table 4:** Contributions of various features in our best closed system (closed-3): decrease in precision/recall/F-measure by leaving one feature at a time.

Feature	P	R	F
Orthographic Feature	27.1	42.5	33.2
POS	23.7	31.1	26.8
Surface Word	12.2	8.1	10.1
Trigger Word	2.4	1.6	1.9
Morphological Pattern	1.3	1.0	1.1
